# Predictive factors for recovery time in patients suffering from severe OHSS

**DOI:** 10.1186/1477-7827-12-59

**Published:** 2014-07-05

**Authors:** Kazem Nouri, Clemens B Tempfer, Christian Lenart, Lisa Windischbauer, Katharina Walch, Regina Promberger, Johannes Ott

**Affiliations:** 1 Department of Obstetrics and Gynecology, Clinical Department of Gynecologic Endocrinology and Reproductive Medicine, Medical University of Vienna, Waehringer Guertel 18-20, 1090 Vienna, Austria; 2 Department of Obstetrics and Gynecology, Ruhr University Bochum, Bochum, Germany; 3 Department of Surgery, Medical University of Vienna, Waehringer Guertel 18-20, 1090 Vienna, Austria

**Keywords:** Long protocol, *In vitro* fertilization, Ovarian hyperstimulation syndrome, Ovulation induction

## Abstract

**Background:**

To evaluate predictive factors for recovery time from severe ovarian hyperstimulation syndrome (OHSS).

**Methods:**

In a retrospective cohort study, 201 women who were hospitalized for severe OHSS were included. Patients with recurrent OHSS were excluded. All the patients received standardized treatment including intravenous hydration, plasma volume expansion, human albumin, furosemid, subcutaneous heparin, and paracentesis if necessary. The main outcome parameter was recovery time from OHSS. Recovery was defined if a morning hematocrit <40%, rebalance of electrolytes, and serum creatinine <1 mg/dL were reached during the standardized therapy and the patient had not suffered from abdominal pain and discomfort at least for one day without any OHSS-specific infusions or medications.

**Results:**

Pregnant patients (n = 80, 39.8%) revealed a longer median duration until recovery than non-pregnant patients (n = 121, 60.2%; 10 days, IQR 7-13, vs. 8 days, IQR 6-10, respectively; p = 0.001). In a generalized linear model, presence of polycystic ovary syndrome before controlled ovarian hyperstimulation (beta = 0.3342 +/- 0.1335, p = 0.012) and use of hCG for ovulation induction (beta = 0.222 +/- 0.1389, p = 0.048) were associated with a longer recovery time in pregnant patients. In non-pregnant patients, none of the tested factors was associated with recovery time.

**Conclusions:**

Pregnant patients with severe OHSS needed a significantly longer recovery time than non-pregnant patients. In pregnant patients, presence of polycystic ovary syndrome and ovulation induction with hCG were associated with longer recovery times.

## Background

Although in vitro fertilization (IVF) has become a safe and common approach to infertility treatment, undesired side effects still remain. Notably, controlled ovarian stimulation (COH) is the main cause of ovarian hyperstimulation syndrome (OHSS).

OHSS is a severe complication caused by COH with gonadotropins. It is characterized by sudden ovarian enlargement with multiple cysts, and an acute shift of intravascular fluid into the third space, leading to ascites, pleural effusion and hemoconcentration, and increased blood viscosity [1]. There are different classifications for OHSS. The most frequently used classification system is the classification by Golan [2]. OHSS occurs in a mild form, which is not of clinical relevance, in up to 30% of all IVF patients [3]. However, 2-3% of all IVF-patients present with a clinically relevant disorder. The incidence of severe OHSS itself is 0.2-1%, which is a life-threatening situation, due to the above-mentioned consequences [4]. Increased hemoconcentration and blood viscosity can cause thrombembolic events that might lead to potentially fatal cardiovascular or neurological events [5]. Although a number of potentially preventive measures have been proposed, complete prevention, especially of late onset OHSS, is still not possible [6].

COH using GnRH agonists (“long protocol”) is still the most commonly used protocol for IVF cycles worldwide. The mechanism of action is pituitary desensitization with subsequent gonadotropin suppression. In order to decrease the risk for OHSS, GnRH agonists were first introduced for triggering the final follicular maturation process (*i.e.* ovulation induction). Thereby, an actual decrease in OHSS incidence could be achieved. However, these can only be used after COH using an antagonist protocol*.* In other words, the use of GnRH agonists (“long protocol”) permits a stronger COH of the ovaries but does not allow the use of GnRH agonists for triggering the final follicular maturation. Hence, this results in an increased incidence of OHSS in patients with long protocol [7-9]. Thus, the use of GnRH antagonists (“antagonist protocol”) is an alternative to GnRH agonists used in long protocol, especially in terms of OHSS prevention [10]. Although the first Cochrane review comparing the two stimulation protocols did not find significant differences in the incidence of OHSS [11], more recent meta-analyses clearly demonstrated that GnRH antagonists were associated with a significant reduction in the incidence of severe OHSS and, thus, the hospitalization rate [12,13]. Nonetheless, the use of GnRH antagonists does not rule out the development of severe OHSS [14]. Indeed, when using GnRH agonists as trigger substances in an antagonist protocol, severe OHSS still can occur [12-14]. Other factors that increase the risk for severe OHSS are presence of polycystic ovary syndrome and a lean body mass [15]. Despite the fact that there is high-level evidence for risk factors for the development of OHSS, as mentioned above, there are few studies about its influence on the course of severe OHSS.

According to recent guidelines, severe OHSS is an indication for hospitalization due to possible life-threatening complications [16]. Usually, patients are released as soon as the patient’s condition has improved and is no longer at high risk. Hence, the time until recovery must be considered a clinically important parameter. This is of clinical impact since, from our experience, patients do suffer decreased quality of life due to OHSS-specific symptoms as well as hospitalization. Moreover, the duration of hospitalization is also of economic relevance. Predictive parameters that would allow assessment of recovery time would be desirable. We, thus, aimed to examine possible influencing factors for the recovery time in severe OHSS. In addition to the type of protocol used for COH and the substance used for ovulation induction, we also focused on other possible influencing parameters.

## Methods

### Patient population

In a retrospective study, all women who underwent COH for IVF at the Department of Gynecologic Endocrinology and Reproductive Medicine of the Medical University of Vienna, Vienna, Austria, from January 1995 until May 2010, and were hospitalized for severe OHSS, were included. All OHSS patients with no reliable information available about the stimulation protocol were excluded from the study. This applied only to women who had undergone COH at another department. In patients who had suffered from severe OHSS more than once, only the first hospitalization was included in the analysis. This resulted in a patient population of 201 women. The study was approved by the ethics committee of the Medical University of Vienna (IRB number 1184/2012).

Severe OHSS was defined as OHSS grade 3 according to Golan and Weissman [2]. Women were hospitalized if they fulfilled at least one criterion of all of the following three groups of OHSS-specific signs:

(i) presence of clinically relevant symptoms, including diffuse abdominal discomfort, nausea, vomiting, dyspnea and tachypnea, subjective decreased urinary output, clinical signs, such as oliguria <600 mL in 24 hours, and/or hypotension;

(ii) electrolyte imbalance, leukocytosis >20,000/mm3, serum creatinine >1 mg/dL, and/or hematocrit >45%; and

(iii) sonographically detectable free fluid (ascites and/or pleural effusion), and a significant increase in ovarian diameter (>12 cm).

The main outcome parameter was recovery time from OHSS and discharge from hospital, which was defined as reaching a morning hematocrit <40%, rebalance of electrolytes, serum creatinine <1 mg/dL, and that the patient had not suffered from abdominal pain and discomfort at least for one day without any OHSS-specific infusions or medications. The above mentioned definition is included in the treatment protocol at our department. These are available in written form to all physicians and they are obliged to adhere to this management scheme.

### Standardized OHSS treatment

We obtained a complete blood count with hematocrit, serum analysis of albumin, blood creatinine, blood urea and creatinine clearance, liver function parameters, and coagulation tests on a daily basis in all patients. The amount of excreted urine, hydric balance, body weight, and abdominal circumference were carefully checked every 24 hours.

In-patient management included bed rest, hydration with 1000 ml lactated Ringer’s solution twice a day; plasma volume expansion with Hydroxyethyl starch solution 6% (HAES-steril® 6% infusion solution, 250 ml KABI, Homburg, Germany), and human albumin 25% (Human Albumin® Octapharm, Langfeld, Germany) once daily in cases of hypoalbuminemia (>34 g/L); and furosemide 20 mg (Lasix® 20 mg, Sanofi, Vienna, Austria), which was administered intravenously in the above-mentioned Ringer’s solution twice a day (resulting in a dose of 40 mg furosemide daily). Paracentesis was performed in patients with ascites that caused pain or compromised pulmonary function (e.g., tachypnea, hypoxia, hydrothorax). A transvaginal approach was used. Directly after the procedure, patients received 100 ml human albumin (see above). Patients had to wear full-length venous support stockings. Prophylactic low-molecular weight heparin therapy (5,000 Units subcutaneously, every 24 hours) was given to all patients during in-patient treatment. From 2008 on, patients were treated in accordance with the recommendations set forth by the Practice Committee of the American Society for Reproductive Medicine in 2008 [1]. In addition to the above mentioned treatment modalities, patients also received 0.5 mg Cabergoline (Dostinex®, Pharmacia, Berlin, Germany) orally once a day for ten days [16] from January 2008. Thus, the inpatient treatment-regimen had not changed during the observation period, apart from introduction of Cabergoline.

Patients were observed for one more day without receiving any infusions or medications when a morning hematocrit <40% was reached for the first time and were usually discharged on the next day if the hematocrit remained <40%. The above mentioned treatment protocol was available in written form to all physicians and they were obliged to adhere to this management scheme, in case of unclearness a telephone hotline with an IVF team member was at all times from in 2001.

### Parameters analyzed

For the first analysis of predictive factors for duration of hospitalization, patients were subdivided in non-pregnant and pregnant patients, since pregnancy is known as a major risk factor for late onset OHSS and it can worsen the medical situation of an early onset OHSS [1,17]. All cases of early onset OHSS that occurred before embryo transfer all patient that had been hospitalized directly after embryo transfer and released home within 10 days, *i.e.* before a possible rise in serum hCG, were also rated as “non-pregnant”. Usually, in case of severe OHSS, embryos would get cryoconserved and transferred in the next cycle. However, in a few cases with low quality of embryos embryo transfer in the same treatment cycle was chosen despite OHSS. This was done only after patients had been informed extensively on their explicit wish. In the second step that included OHSS-specific parameters, pregnant and non-pregnant women were not analyzed separately.

We analyzed the type of stimulation protocol (long protocol vs. other protocols) and the substance used for ovulation induction (human chorionic gonadotropin, hCG, vs. other substances). We also included the type of stimulation protocol as a predictive parameter in addition to the use of hCG for ovulation induction, since the fact that GnRH antagonist cycles have a lower risk of OHSS is also due to differences in follicular recruitment. Accordingly, even when inducing ovulation with hCG, there is a significant reduced risk of OHSS in antagonist cycles as compared to agonist cycles [18]. For agonist triggering, 0.2 mg Triptorelinacetate (Decapeptyl ®, Ferring, Germany) had been used in patients in danger of developing OHSS in case of >15 follicles in each ovary.

In addition, we also focused on the following parameters as possible predictive factors for the recovery time: age; body mass index (BMI); presence of polycystic ovary syndrome that was known to be associated with a higher incidence of OHSS [19]; number of retrieved oocytes; time between oocyte retrieval and hospitalization for OHSS; whether the patient suffered from early or late onset OHSS; the use of Cabergoline; and the year the patient was treated for OHSS (years 1996-2007, i.e. before the new treatment recommendations [1] were published and implemented in daily routine at our department vs. 2008-2010, i.e. afterwards) in order to rule out any additional bias due to the introduction of these recommendations [1] despite the fact that the standardized treatment regimen had not changed at our department. In the group of pregnant patients, we also included multiple pregnancies as a predictive factor. Since standard luteal support included only dydrogesterone and/or progesterone supplementation and, in no case, hCG was used for luteal support, we did not have to evaluate the latter as a predictive factor for recovery time.

In a next step, we also included the following OHSS-specific parameters in the model: the initial hematocrit at the time of hospitalization and the maximum hematocrit seen in the course of regular, daily blood count controls and the need of paracentesis.

### Statistical analysis

Nominal variables are reported as numbers and frequencies, and continuous variables as medians and interquartile ranges (IQR). Statistical analysis was accomplished using Wilcoxon rank-sum tests where applicable and generalized linear models with a Poisson link function. For the latter, coefficient estimates β and eβ, 95%-confidence interval (CI), standard error se(β), and corresponding p-values are given. Differences between groups were tested using the ANOVA for numeric variables and the Chi square test or the Fisher’s exact test for categorical variables where appropriate. P-values <0.05 were considered statistically significant. Statistical analyses were performed with the SPSS software package, version 19 (SPSS, Chicago).

## Results

Details about basic patient characteristics are provided in Table 1. In 156 patients (77.2%), hCG had been used for ovulation induction (10000 I.E of urinary hCG (Pregnyl ® Organon, Holland) in 147 cases and 6500 I.E. of recombinant hCG (Ovitrel 250 mg ®Merk, UK) in nine cases). None of the patients had complications such as venous thrombosis or renal insufficiency. The median recovery time was 9 days (IQR 7-11 days). Pregnant patients (n = 80, 39.8%) revealed a significantly longer median duration of hospitalization than non-pregnant patients (n = 121, 60.2%; 10 days, IQR 7-13, vs. 8 days, IQR 6-10, respectively; p = 0.001).

**Table 1:** General patient characteristics

**Number of patients**	**201**
*Age (years)*^a^	30 (26-34)
*Body mass index (kg/m*^*2*^*)*^a^	22.4 (20.9-26.1)
*Tubal factor*^b^	37 (18.4)
*Male factor*^b^	143 (71.1)
*Endometriosis*^b^	7 (3.5)
*Polycystic ovary syndrome*^b^	19 (9.5)
*Idiopathic infertility*^b^	15 (7.5)
*Use of long protocol for controlled ovarian hyperstimulation*^b^	94 (46.5)
*Use of hCG for ovulation induction*^b^	156 (77.2)
*Number of retrieved oocytes (n)*^a^	12 (9-17)
*Time between oocyte retrieval and hospitalization for OHSS (days)*^a^	4 (3-8)

Data are provided as ^a^median (interquartile range) or ^b^n (%).

Multiple citations possible.

In a generalized linear model, we calculated factors predictive for the recovery time in pregnant patients (Table 2). Presence of polycystic ovary syndrome before COH (ß = 0.322 ± 0.1326, p = 0.015) and use of hCG for ovulation induction (ß = 0.238 ± 0.1373, p = 0.043) were associated with a longer duration of hospitalization. The median duration of hospitalization differed significantly between women with without polycystic ovary syndrome before (median 11 days, IQR 7-14, vs. median 9 days, IQR 7-12, respectively; p = 0.044) as well as between the types of ovulation induction (hCG: median 10 days, IQR 8-14, vs. GnRH agonists: median 9 days, IQR 7-12; p = 0.047). These results differed from the findings in non-pregnant patients: in the latter, none of the tested factors was associated with the duration of hospitalization (Table 2).

**Table 2:** Generalized linear model using a Poisson link function to predict the recovery time from severe OHSS

**Parameter**	***Pregnant patients (n = 80)***					***Non-pregnant patients (n = 121)***				
	**ß**	**Standard error (ß)**	**95% CI**		**p**	**ß**	**Standard error (ß)**	**95% CI**		**p**
			**Lower**	**Upper**				**Lower**	**Upper**	
*(Intercept)*	1.061	0.5915	-0.099	2.220	0.073	2.425	0.5608	1.326	3.542	**<0.0001**
*Use of cabergoline*	-0.182	0.3329	-0.834	0.471	0.585	-0.013	0.0918	-0.193	0.167	0.890
*Late onset OHSS*	0.282	0.2364	-0.181	0.746	0.233	-0.009	0.2914	-0.580	0.562	0.976
*Polycystic ovary syndrome*	0.334	0.1335	0.073	0.596	**0.012**	-0.025	0.1528	-0.274	0.325	0.868
*Use of hCG for ovulation induction*	0.222	0.1389	0.002	0.495	**0.048**	0.015	0.0941	-0.169	0.200	0.872
*Number of retrieved oocytes (n)*	0.015	0.0089	-0.002	0.033	0.084	0.004	0.0072	-0.010	0.018	0.603
*Year of COH/OHSS treatment: 1996-2007*	0.528	0.3666	-0.190	1.247	0.149	-0.071	0.0984	-0.261	0.128	0.482
*Use of long protocol*	0.053	0.1263	-0.194	0.301	0.673	-0.047	0.1014	-0.246	0.151	0.641
*Age (years)*	0.013	0.0110	-0.008	0.035	0.230	-0.003	0.0088	-0.020	0.014	0.740
*Body mass index (kg/m*^*2*^*)*	0.008	0.0086	-0.009	0.025	0.360	-0.006	0.0102	-0.026	0.014	0.585
*Multiple pregnancy (n)*	0.195	0.1077	-0.016	0.406	0.060	**-**	**-**	**-**	**-**	**-**
*Time between oocyte retrieval and hospitalization for OHSS (days)*	0.009	0.0180	-0.027	0.044	0.636	-0.020	0.0212	-0.061	0.022	0.353

Coefficient estimates β, standard error se(β), 95%-confidence interval (CI) = β ± z^α/2^ se(β), and corresponding p-value are summarized in the table. The categorical factors are compared to the missing category, *i.e.*, no use of cabergoline, early onset OHSS, no polycystic ovary syndrome, use of other substances than hCG analogues for ovulation induction, year of treatment 2008-2010, controlled ovarian hyperstimulation with other protocols than long protocol, and no multiple pregnancy. In the analysis of non-pregnant patients, the parameter “year of treatment” had to be excluded for this analysis, since it was redundant to the parameter “use of long protocol”. Statistically significant p-values are printed in bold numbers.

In a next step, we included OHSS-specific parameters in the generalized linear model (Table 3). At the time of hospitalization, the initial hematocrit level was 42.3% (IQR 39.9-45.7). The median maximum hematocrit level (45.6%, IQR 42.9-47.9) was reached after a median of 1 day (IQR 1-3; range 1-10). Fifty-one women (25.4%) were in need for paracentesis due to severe abdominal pain or compromised pulmonary function. When also including these OHSS-specific markers into a multivariate model on all patients, pregnancy (ß = 0.163 ± 0.0756, p = 0.031), presence of polycystic ovary syndrome (ß = 0.332 ± 0.1065, p = 0.002), use of hCG for ovulation induction (ß = 1.74 ± 0.0804, p = 0.030), need for paracentesis (ß = 0.446 ± 0.0795, p < 0.001) and both the initial (ß = 0.049 ± 0.0198, p = 0.013) and maximum hematocrit level (ß = 0.048 ± 0.0200, p = 0.016) were significantly predictive for the recovery time of OHSS.

**Table 3:** Generalized linear model using a Poisson link function to predict the recovery time from severe OHSS: general and OHSS-specific factors

**Parameter**	**ß**	**Standard error (ß)**	**95% CI**		**p**
			**Lower**	**Upper**	
*(Intercept)*	1.758	0.5485	0.683	2.833	**0.001**
*Pregnancy*	0.163	0.0756	0.015	0.312	**0.031**
*Use of dostinex*	-0.490	0.3169	-1.111	0.131	0.122
*Early (vs. late) onset OHSS*	0.206	0.1785	-0.144	0.555	0.249
*Polycystic ovary syndrome*	0.332	0.1065	0.124	0.541	**0.002**
*Use of long protocol*	0.051	0.0753	-0.096	0.199	0.497
*Number of retrieved oocytes (n)*	0.003	0.0056	-0.008	0.018	0.630
*Year of COH/OHSS treatment: 1996-2007*	0.655	0.3262	-0.115	1.294	0.065
*Use of hCG for ovulation induction*	0.174	0.0804	-0.017	0.332	**0.030**
*Age*	0.005	0.0065	-0.008	0.018	0.422
*BMI*	0.010	0.0069	-0.004	0.023	0.159
*Time between oocyte retrieval and hospitalization for OHSS (days)*	-0.005	0.0137	-0.032	0.022	0.696
*Necessity for paracentesis*	0.446	0.0795	0.290	0.602	**<0.001**
*Initial hematocrit (%)*	0.049	0.0198	0.010	0.088	**0.013**
*Maximum hematocrit (%)*	0.048	0.0200	0.009	0.088	**0.016**

Coefficient estimates β, standard error se(β), 95%-confidence interval (CI) = β ± z^α/2^ se(β) , and corresponding p-value are summarized in the table. The categorical factors are compared to the missing category, *i.e.*, no use of cabergoline, early onset OHSS, no polycystic ovary syndrome, use of other substances than hCG analogues for ovulation induction, year of treatment 2008-2010, controlled ovarian hyperstimulation with other protocols than long protocol, and no need for paracentesis. Statistically significant p-values are printed in bold numbers.

## Discussion

In this retrospective study, presence of polycystic ovary syndrome and the use of hCG for ovulation induction were found to be independently associated with a longer recovery time in pregnant patients with severe OHSS. Notably, none of the tested parameters was predictive for the duration of recovery in non-pregnant patients. In our data set, the median recovery time was nine days. Of course, this is dependent on the definition of recovery. In our study, recovery was defined when a hematocrit of <40%, electrolyte balance, a normal leucocyte count and serum creatinine <1 mg/dl were reached, patients did not suffer any longer from clinically relevant symptoms and this situation persisted after one day without any OHSS-specific treatment. One might consider these criteria strict since a hematocrit >45% defines severe OHSS of grade 3 [2]. We consider the a hematocrit cut-off of 45% for the definition of OHSS recovery as not useful, since the definition of severe OHSS is not based on hematocrit alone and many patients do not reveal hematocrit levels >45% at the time at diagnosis. This is also in accordance with our results (median initial hematocrit 42.3%, median maximum hematocrit level 45.6%). To date, an exact definition of recovery has not been provided in the literature. For example, in their 2008 recommendations, the Practice Committee of the American Society for Reproductive Medicine only stated that “serial clinical and laboratory evaluations provide the means to recognize evidence of resolution”, but do not focus on recovery any further [1]. In addition, there is also a lack of data on recovery time from severe OHSS in the literature.

As demonstrated in Table 2, presence of PCOS was a significant factor predictive of recovery time in pregnant patients. PCOS is a well-known risk factor for the development of OHSS [15,17]. It is noteworthy that, in our patient population, it also extended the course of severe OHSS by a median of two days.

The second predictive factor in these patients was the use of hCG for ovulation induction. Traditionally, in COS, hCG is administered as a surrogate for the natural LH surge to induce final oocyte maturation. Since hCG has a longer half life compared with LH, it has been hypothesized that it could extend the luteinizing stimulus for the granulosa cells and, thereby, could play a crucial role in the development of OHSS [19]. While the luteinizing stimulus exerted by exogenous hCG is the major driving force behind early OHSS that starts within eight days after oocyte retrieval, late OHSS seems to be triggered by endogenous. In case that a patient is at an increased risk of OHSS, withholding the administration of hCG and aborting the cycle of COH can prevent the patient from either form of the syndrome [20].

More recently, the use of a GnRH agonist instead of hCG for ovulation induction has been proposed as a preventive method to avoid the appearance of OHSS. The limitation of this measure is the fact that it can only be achieved in COS cycles in which a GnRH antagonist is used for pituitary suppression. GnRH agonists used in this context would have two functions: first, inducing final oocyte maturation; and second, acting as a luteolytic agent and thereby preventing the secretion of vasoactive substances, namely Vascular Endothelial Growth Factor (VEGF) from corpora lutea [21]. Our data support this finding and could also demonstrate that in pregnant women who suffered from severe OHSS, the use of hCG as a trigger substance for final follicular maturation was associated with a longer recovery time, extending it by a median of one day.

Notably, the type of stimulation protocol was not predictive for recovery time. This is somehow in line with the fact that, concerning the development of OHSS, (i) the use of GnRH agonists for ovulation induction after COH with an antagonist protocol can prevent the patient from OHSS and (ii) that the use of a “long protocol” leads more likely to OHSS since ovulation cannot be induced with the “low-risk substances”, *i.e.* GnRH agonists [19-21]. Accordingly, the substance used for ovulation induction is of a higher overall impact than the stimulation protocol itself and this also seems to be the case for the duration of severe OHSS.

In this study, we confirm that PCOS and hCG are significant contributors to severe OHSS. Notably, in non-pregnant patients, neither presence of PCOS and ovulation induction with hCG nor the other patient- and IVF-related factors were predictive for the duration of hospitalization. One has to take into account that pregnancy is the main factor for late onset OHSS and that the pregnancy rate is higher in patients with fresh embryo transfer after hCG compared to GnRH agonist triggering. This likely contributes to the differences in predictive factors for duration of OHSS.

As recently reviewed, pregnancy substantially increases the risk for OHSS and this is thought to be due to an increase in endogenous hCG [17]. Vascular endothelial growth factor (VEGF) has been identified as the major mediator of OHSS [17]. With the exogenous administration of hCG, the expression of VEGF increases significantly and the maximum rise coincides with peaked vascular permeability [22]. Hypothetically spoken, assuming that ovulation induction with hCG would only be a short term stimulus for VEGF expression in non-pregnant patients, the absence of a predictive value of hCG administration for OHSS recovery time in these women seems reasonable. In other words, the endogenous hCG production in pregnant OHSS patients could sustain VEGF expression that had initially been increased with administration of hCG for ovulation induction. In addition, increased VEGF levels have been documented in women with PCOS [23,24]. One could hypothesize that this might have contributed to the prolonged recovery time from OHSS in patients with PCOS. However, this remains completely speculative.

It should be emphasized that only women who already suffered from severe OHSS were included in our analysis. This might contribute to the fact that other parameters that are known to influence the incidence of severe OHSS such as the time interval between stimulation and onset of OHSS (late vs. early OHSS) were not associated with recovery time [25,26]. Another notable result of our study was the fact that the introduction of Cabergoline treatment did not lead to a significant reduction in recovery times from severe OHSS. Recently, it has been suggested that the use of dopamine agonists appeared to be effective for the prevention of OHSS, but was less effective for the treatment of OHSS [27,28]. We, however, present only non-randomized data. Notably, the OHSS-specific parameters higher initial and maximum hematocrit levels as well as the patients’ need for paracentesis were also reliably predictive for longer recovery times (Table 3) and, thus, can also be used to assess recovery time in an individual patient.

One might argue that diuretics such as furosemide are actually contraindicated in OHSS patients due to known intravascular volume depletion. Hypothetically spoken, in case of OHSS, furosemide could put the patient at risk for further hemoconcentration. However, it also offers the advantage of preventing oliguria/anuria, which is also a feared complication of OHSS. Therefore we suggest using furosemide only after having started with the correction of intravascular volume and rehydration of the OHSS patient. The latter is guaranteed in our treatment regimen, since furosemide is only administered in combination with 2,000 ml of intravenous saline infusion/day. In contrast to the recommendations of Alper et al., we do not recommend reduction of oral fluid intake to the patients [29]. One might say that our aim is to reach both a high volume of fluid in- and output. We do this in order to correct intravascular hemoconcentration and also prevent renal failure. We are also aware of the recommendation to use furosemide only after the hematocrit level has decreased to 38% or lower (1). However, there was no case of thromboembolism and also no case of renal insufficiency in our study population over a long period of 15 years. Thus, we consider our regimen including routine furosemide treatment safe. In addition to these considerations, we hazarded the hypothetical consequences of the fact that furosemide is a FDA category C drug which means that risk cannot be ruled out during pregnancy.

The retrospective study design, our specific definition of OHSS recovery and the treatment including furosemide have to be considered limitations of this report. In order to guarantee sound data it is of high relevance that the decision on patients’ discharge was based on the same criteria by all the doctors. This might have been a clinical problem, since on weekends doctors from other divisions within the Department of Obstetrics and Gynecology were on duty. Therefore, a telephone hotline with an IVF team member available for questions regarding OHSS patients at all times had been instituted in 2001. We are thus confident that the management was consistent even on weekends and public holidays, at least since 2001. One might wonder why PCOS patients, known to be at a high risk for OHSS, had been stimulated using a GNRH agonist protocol. This was the case only in the early years of this historic patient population (i.e. 1995-2010), since the so called long protocol had been the dominating treatment in this time period. Moreover, we consider that the type of administered hCG (urinary versus recombinant) might have also influenced the duration of OHSS. However, due to the small number of cases with recombinant hCG, we could not analyse this factor separately. One also has to consider that, in the initial study period, cabergoline had not been given as a primary prophylactic treatment for women at risk for OHSS. We cannot rule out that cabergoline use versus non-use may have affected the characteristics of the study population. In this regard, it is possible that cabergoline use, which is a physician choice, could have affected population characteristics such as age or BMI. This might also be a study limitation. Nonetheless, this is the first study to evaluate the factors that influence the recovery time from severe OHSS. We are aware of the fact that our study does not add to the knowledge about prevention of OHSS. We, nonetheless, consider it of interest to learn about predictive factors for recovery time due to its economic and also medical implications.

Discharge from hospital is dependent on the patients’ condition and, thus, on improvement of OHSS symptoms. OHSS patients are only released from hospital when they are no longer at high risk. It is obvious that during the time of hospitalisation patients experience decreased quality of life due to OHSS-specific symptoms and treatment which may also include repeated paracentesis. Hence, the time until recovery must be considered a clinically important parameter. Moreover, the duration of hospitalization likely is of economic relevance. Our study provides (i) a definition of recovery from OHSS for the first time; (ii) parameters that predict recovery time from OHSS which might facilitate counselling of patients who usually wish for their release home; and (iii) we share our 15-year experience with our management of severe OHSS.

## Conclusions

Notably, only in pregnant patients, presence of PCOS and ovulation induction with hCG were found to have led to longer recovery times. OHSS-specific parameters allow a more reliable prediction of recovery time. Further studies that focus on recovery to confirm our results are warranted. In addition, a consensus on the definition of OHSS recovery would be desirable, probably not only from a clinical, but also from a forensic point of view.

## Competing interests

All authors declare that there are no potential competing interest, whether of a financial or other nature.

## Authors’ contributions

All authors contributed to the writing process of the manuscript and approved the final version. KN and JO were the principal investigators, wrote the study protocol and manuscript. CL, LW, KW,CT and RP worked as co-investigators performed the literature search and were crucially involved in data interpretation. All authors read and approved the final manuscript.
